# Syntheses and crystal structures of two adamantyl-substituted 1,2,4-triazole-5-thione *N*-Mannich bases

**DOI:** 10.1107/S2056989017009756

**Published:** 2017-07-07

**Authors:** Monirah A. Al-Alshaikh, Aamal A. Al-Mutairi, Hazem A. Ghabbour, Ali A. El-Emam, Mohammed S. M. Abdelbaky, Santiago Garcia-Granda

**Affiliations:** a Department of Chemistry, College of Sciences, King Saud University, Riyadh 11451, Saudi Arabia; b Department of Pharmaceutical Chemistry, College of Pharmacy, King Saud University, Riyadh 11451, Saudi Arabia; c Department of Physical and Analytical Chemistry, Faculty of Chemistry, Oviedo University–CINN, Oviedo 33006, Spain

**Keywords:** crystal structure, 1,2,4-triazole-5-thione, *N*-Mannich bases, adamantane, piperazine, biological activity, C—H⋯F hydrogen bonding: C—H⋯π inter­actions

## Abstract

In the title *N*-Mannich bases, 3-(adamantan-1-yl)-4-(4-fluoro­phen­yl)-1-[(4-phenyl­piperazin-1-yl)meth­yl]-4,5-di­hydro-1*H*-1,2,4-triazole-5-thione (C_29_H_34_FN_5_S) (I), and 3-(adamantan-1-yl)-4-(4-fluoro­phen­yl)-1-{[4-(2-meth­oxyphen­yl)piperazin-1-yl]-meth­yl}-4,5-di­hydro-1*H*-1,2,4-triazole-5-thione (C_30_H_36_FN_5_OS) (II), fluoro­phenyl, adamantane and piperazine moieties are linked to a planar triazole ring. There is an additional phenyl ring on the piperazine ring in (I) and a meth­oxy­phenyl ring in (II). In compound (I), the fluoro­phenyl and phenyl rings are inclined to the triazole ring by 86.55 (13) and 60.52 (12)°, respectively, and the two aryl rings are inclined to one another by 66.37 (13)°. In compound (II), the corresponding dihedral angles are 83.35 (13), 71.38 (15) and 11.97 (16)°, respectively. The crystal structure of (I) shows pairs of C—H⋯F hydrogen bonds forming inversion dimers, while in the crystal of compound (II), in addition to the C—H⋯F hydrogen bonds that generate chains parallel to the *b* axis, there are C—H⋯π inter­actions present that link the chains to form layers parallel to the *ab* plane.

## Abstract 2

The syntheses and crystal structures of two novel adamantane-substituted *N*-Mannich bases are reported herein. In the crystals of both compounds, there are C—H⋯F hydrogen bonds present, forming inversion dimers in one and chains in the other.

## Chemical context   

The incorporation of an adamantyl moiety into various bioactive mol­ecules results in compounds with relatively high lipophilicity, which in turn modifies the bioavailability and modulates the therapeutic indices through various mechanisms (Liu *et al.*, 2011 ▸; Lamoureux & Artavia, 2010 ▸). Several adamantane-based drugs have been developed as anti­viral (Davies *et al.*, 1964 ▸; Togo *et al.*, 1968 ▸; Rosenthal *et al.*, 1982 ▸; El-Emam *et al.*, 2004 ▸; Burstein *et al.*, 1999 ▸; Balzarini *et al.*, 2009 ▸), anti-Parkinsonian (Schwab *et al.*, 1969 ▸) and hypoglycaemic (Villhauer *et al.*, 2003 ▸; Augeri *et al.*, 2005 ▸) drugs. In addition, numerous adamantane-based analogues have promising anti­cancer (Sun *et al.*, 2002 ▸), bactericidal (Protopopova *et al.*, 2005 ▸; El-Emam *et al.*, 2013 ▸; Kadi *et al.*, 2010 ▸; Al-Abdullah *et al.*; 2014 ▸; Al-Deeb *et al.*, 2006 ▸) and fungicidal (Omar *et al.*, 2010 ▸) activities. In a continuation of our ongoing studies on the pharmacological and structural properties of adamantyl 1,2,4-triazole derivatives (Al-Abdullah *et al.*, 2012 ▸; Al-Tamimi *et al.*, 2014 ▸; El-Emam *et al.*; 2013 ▸; 2014 ▸), we report herein on the synthesis and crystal structures of the title adamantyl-substituted 1,2,4-triazole-5-thione *N*-Mannich bases, (I) and (II).
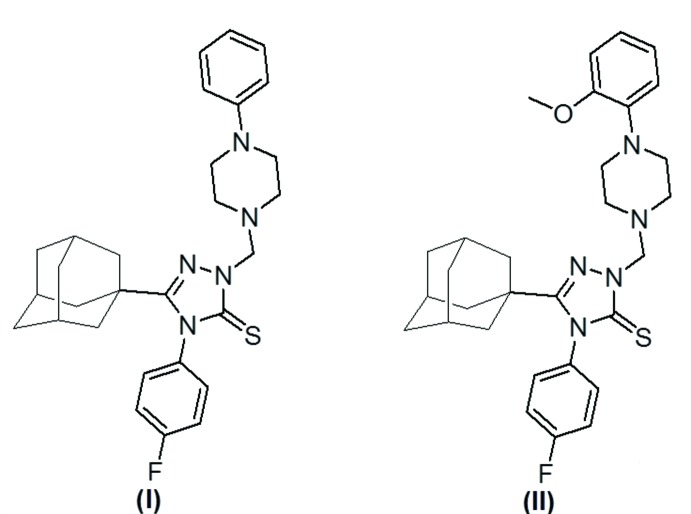


## Structural commentary   

**Figure 1:**
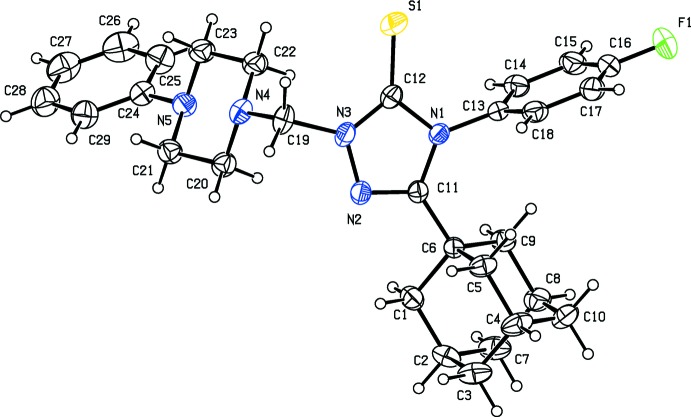
The mol­ecular structure of compound (I), with the atom labelling and 30% probability displacement ellipsoids.

**Figure 3:**
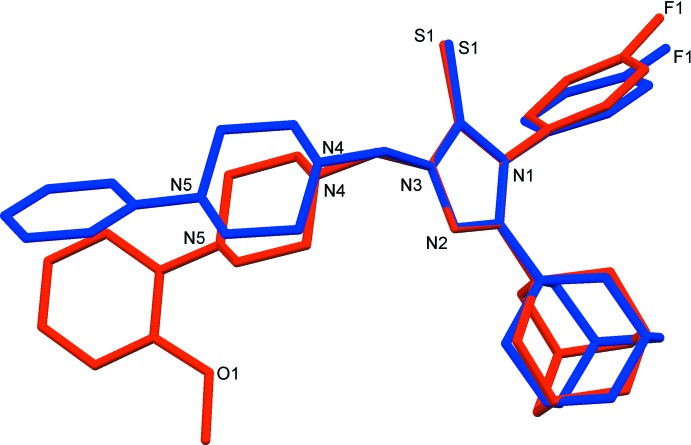
A structural overlap of compounds (I) and (II) [colour code: (I) blue, (II) red].

The mol­ecular structures of the title compounds, (I) and (II), are illustrated in Figs. 1 ▸ and 2 ▸, respectively. In both mol­ecules the piperazine rings have a chair conformation, with the N-bound substituents occupying equatorial positions. In (I), the fluoro­phenyl ring (C13–C18) and the phenyl ring (C24–C29) are inclined to the triazole ring (N1–N3/C11/C12) by 86.55 (13) and 60.52 (12)°, respectively. The two aryl rings are inclined to one another by 66.37 (13)°. In compound (II), the fluoro­phenyl ring (C13–C18) and the phenyl ring (C24–C29) are inclined to the triazole ring (N1–N3/C11/C12) by 83.35 (13) and 71.38 (15)°, respectively, while the two aryl rings are inclined to one another by only 11.97 (16)°. This difference in conformation is illustrated by the structural overlap diagram, shown in Fig. 3 ▸.

## Supra­molecular features   

**Figure 4:**
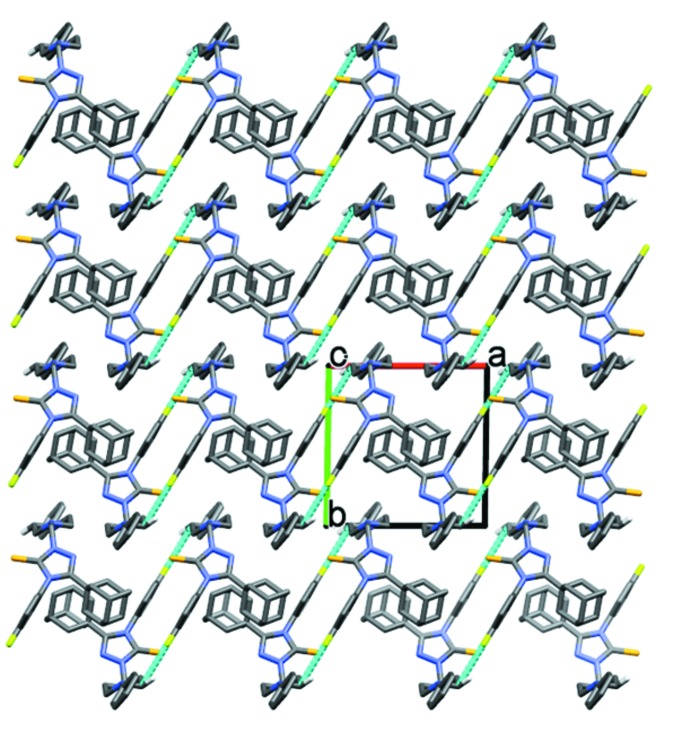
The crystal packing of compound (I), viewed along the *c* axis. The hydrogen bonds are shown as dashed lines (see Table 1 ▸), and only the H atoms involved in these inter­actions have been included.

**Figure 5:**
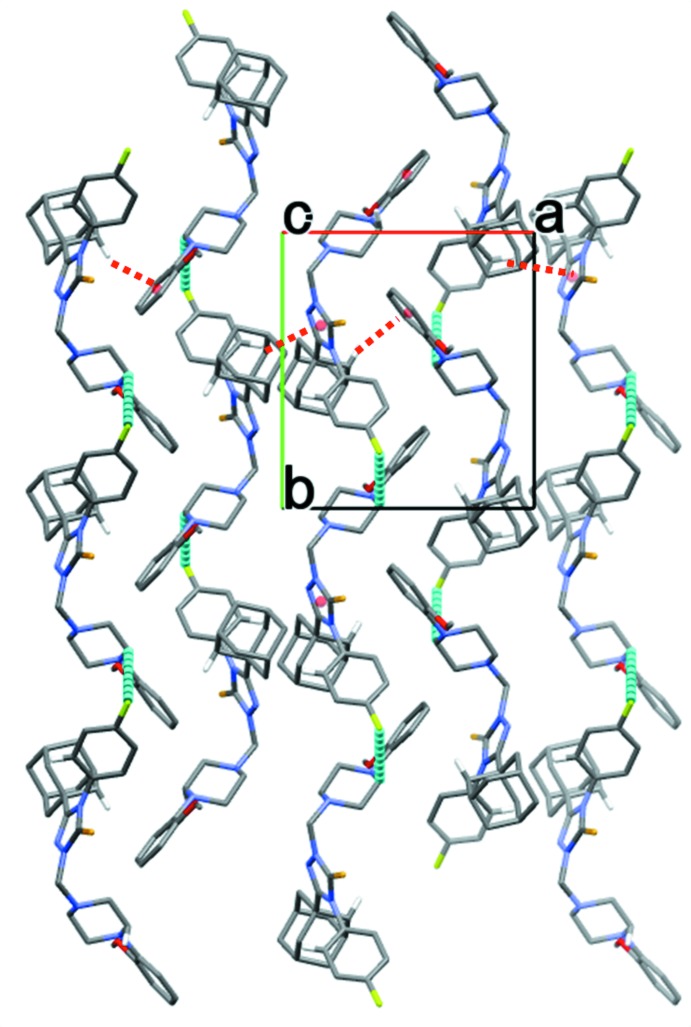
The crystal packing of compound (II), viewed along the *c* axis, showing the C—H⋯F hydrogen bonds (dashed cyan lines) and some of the C—H⋯π inter­actions (dashed red lines); see Table 2 ▸. Only the H atoms involved in these inter­actions have been included.

In the crystal of compound (I), mol­ecules are linked by pairs of C—H⋯F hydrogen bonds, forming inversion dimers (Table 1 ▸ and Fig. 4 ▸). In compound (II), mol­ecules are linked by C—H⋯F hydrogen bonds, forming chains parallel to the *b-*axis direction. The chains are linked by C—H⋯π inter­actions, forming layers parallel to the *ab* plane (Table 2 ▸ and Fig. 5 ▸).

## Database survey   

A search of the Cambridge Structural Database (Version 5.38, last update May 2017; Groom *et al.*, 2016 ▸) for the 3-(adamantan-1-yl)-4-[(piperazin-1-yl) meth­yl]-1,2,4-triazole-3(3*H*)-thione moiety gave 14 hits. One compound, 3-(adamantan-1-yl)-4-phenyl-1-[(4-phenyl­piperazin-1-yl)meth­yl]-1*H*-1,2,4-triazole-5(4*H*)-thione (GAPWUR; Al-Abdullah *et al.*, 2012 ▸), is very similar to compound (I). It has a phenyl ring substituent on the piperazine ring and a phenyl ring substituent on the triazole ring, which are inclined to one another by 72.4 (2)°, and by 89.0 (2) and 74.4 (2)°, respectively, to the triazole ring. In compound (I), the corresponding dihedral angles are 66.37 (13), 86.55 (13) and 60.52 (12)°, respectively. Two compounds have a 2-meth­oxy­phenyl ring substituent on the piperazine ring, *viz.* (3-(1-adamant­yl)-1-{[4-(2-meth­oxy­phen­yl)piperazin-1-yl]meth­yl}-4-methyl-1*H*-1,2,4-triazole-5(4*H*)-thione (YUPVIP; El-Emam *et al.*, 2014 ▸), with a methyl substituent on the triazole ring, and 3-(adamantan-1-yl)-4-ethyl-1-{[4-(2-meth­oxy­phen­yl)piperazin-1-yl]meth­yl}-1*H*-1, 2,4-triazole-5(4*H*)-thione (RITMUE; Al-Tamimi *et al.*, 2010 ▸) with an ethyl substituent on the triazole ring. In these two compounds the meth­oxy­phenyl rings are inclined to the triazole ring by 67.1 (1) and 59.4 (1)°, respectively. In compound (II), the corresponding dihedral angle is 71.38 (15)°.

## Synthesis and crystallization   

**Figure 6:**
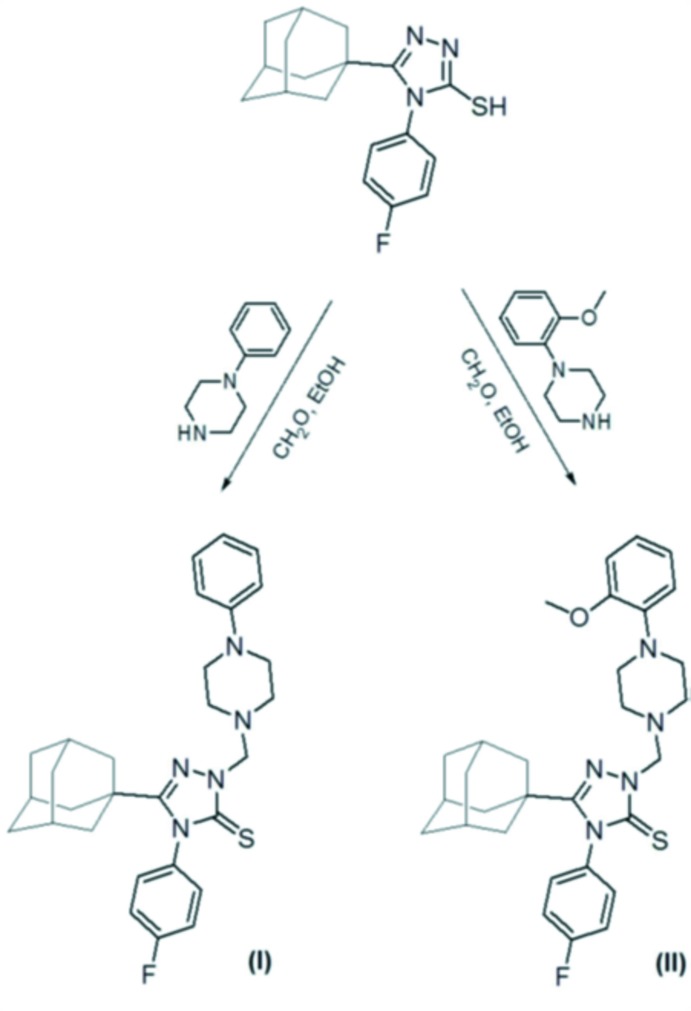
Reaction schemes for the syntheses of compounds (I) and (II).

The title compounds, (I) and (II), were synthesized *via* the reaction of 3-(adamantan-1-yl)-4-(4-fluoro­meth­yl)-4*H*-1,2,4-triazole-5-thiol (Al-Deeb *et al.*, 2006 ▸) with the appropriate monosubstituted piperazie and a formaldehyde solution, as outlined in Fig. 6 ▸.

**Compound (I)**: 1-Phenyl­piperazine (325 mg, 2 mmol) and a 37% formaldehyde solution (1 ml) were added to a solution of 3-(adamantan-1-yl)-4-(4-fluoro­meth­yl)-4*H*-1,2,4-triazole-5-thiol (659 mg, 2 mmol) in ethanol (10 ml), and the mixture was heated under reflux for 1 h then allowed to stand overnight. Cold water (3 ml) was slowly added and the mixture was stirred for 20 min. The precipitated crude product was filtered, washed with water, dried, and crystallized from ethanol to yield 846 mg (84%) of compound (I) as colourless plate-shaped crystals (m.p. 469–471 K). ^1^H NMR (700.17 MHz): δ 1.47–1.49 (*m*, 3H, adamantane-H), 1.60–1.62 (*m*, 3H, adamantane-H), 1.80 (*s*, 6H, adamantane-H), 1.89 (*s*, 3H, adamantane-H), 2.89–2.91 (*m*, 4H, piperazine-H), 3.14–3.15 (*m*, 4H, piperazine-H), 5.14 (*s*, 2H, CH_2_), 6.77–6.79 (*m*, 1H, Ar-H), 6.94 (*d*, 2H, Ar-H, *J* = 8.4 Hz), 7.20–7.22 (*m*, 2H, Ar-H), 7.41–7.49 (*m*, 4H, Ar-H). ^13^C NMR (125.76 MHz): δ 27.61, 36.07, 39.62, 39.74 (adamantane-C), 48.73, 50.30 (piperazine-C), 69.06 (CH_2_), 116.0, 116.78, 119.41, 129.37, 132.80, 133.10, 151.48, 156.28 (Ar-C), 162.17 (triazole C-3), 170.95 (C=S).

**Compound (II)**: 1-(2-Meth­oxy­phen­yl)piperazine (385 mg, 2 mmol) and a 37% formaldehyde solution (1 ml) were added to a solution of 3-(adamantan-1-yl)-4-(4-fluoro­meth­yl)-4*H*-1,2,4-triazole-5-thiol (659 mg, 2 mmol) in ethanol (10 ml), and the mixture was heated under reflux for 1 h then allowed to stand overnight. The precipitated crude product was filtered, washed with cold ethanol, dried, and crystallized from ethanol to yield 865 mg (81%) of compound (II) as colourless block-like crystals (m.p. 462–464 K). ^1^H NMR (700.17 MHz): δ 1.49–1.50 (*m*, 3H, adamantane-H), 1.61–1.63 (*m*, 3H, adamantane-H), 1.83 (*s*, 6H, adamantane-H), 1.90 (*s*, 3H, adamantane-H), 2.89–2.90 (*m*, 4H, piperazine-H), 2.96–2.98 (*m*, 4H, piperazine-H), 3.78 (*s*, 3H, OCH_3_), 5.11 (*s*, 2H, CH2), 6.88–6.96 (*m*, 4H, Ar-H), 7.42–7.52 (*m*, 4H, Ar-H). ^13^C NMR (125.76 MHz): δ 27.61, 35.77, 36.07, 39.61 (adamantane-C), 50.54, 50.58 (piperazine-C), 55.65 (OCH_3_), 69.39 (CH_2_), 112.08, 116.78, 118.51, 121.23, 123.02, 132.79, 133.13, 141.53, 152.36, 156.32 (Ar-C), 162.17 (triazole C-3), 171.0 (C=S).

Suitable single crystals of compounds (I) and (II) were obtained by slow evaporation of CHCl_3_:EtOH solutions (1:1, 5 ml) at room temperature.

## Refinement   

Crystal data, data collection and structure refinement details are summarized in Table 3 ▸.The C-bound H atoms were positioned geometrically and treated as riding atoms: C—H = 0.93–1.00 Å with *U*_iso_(H) = 1.5*U*_eq_(C-meth­yl) and 1.2*U*_eq_(C) for other H atoms.

## Supplementary Material

Crystal structure: contains datablock(s) global, I, II. DOI: 10.1107/S2056989017009756/su5368sup1.cif

Structure factors: contains datablock(s) I. DOI: 10.1107/S2056989017009756/su5368Isup2.hkl

Structure factors: contains datablock(s) II. DOI: 10.1107/S2056989017009756/su5368IIsup3.hkl

Supporting information file. DOI: 10.1107/S2056989017009756/su5368Isup4.cml

Supporting information file. DOI: 10.1107/S2056989017009756/su5368IIsup5.cml

CCDC references: 1559732, 1559731

Additional supporting information:  crystallographic information; 3D view; checkCIF report

## Figures and Tables

**Figure 2:**
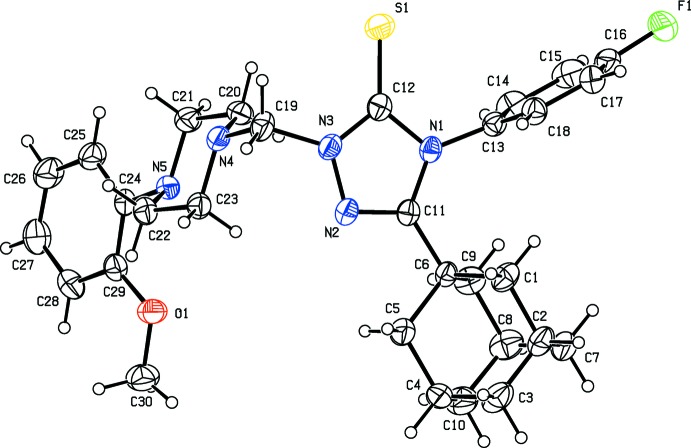
The mol­ecular structure of compound (II), with the atom labelling and 30% probability displacement ellipsoids.

**Table 1:** Hydrogen-bond geometry (Å, °) for (I)

*D*—H⋯*A*	*D*—H	H⋯*A*	*D*⋯*A*	*D*—H⋯*A*
C22—H22*B*⋯F1^i^	0.99	2.49	3.332 (4)	142

Symmetry code: (i) 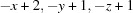.

**Table 2:** Hydrogen-bond geometry (Å, °) for (II) *Cg*1 and *Cg*8 are the centroids of rings (N1–N3/C11/C12) and (C24–C29), respectively.

*D*—H⋯*A*	*D*—H	H⋯*A*	*D*⋯*A*	*D*—H⋯*A*
C21—H21*A*⋯F1^i^	0.97	2.47	3.407 (3)	162
C18—H18*A*⋯*Cg*1^ii^	0.93	2.81	3.661	152
C9—H9*A*⋯*Cg*8^iii^	0.97	2.80	3.697	155

Symmetry codes: (i) 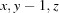; (ii) 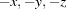; (iii) 
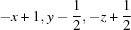
.

**Table 3:** Experimental details

	(I)	(II)
Crystal data		
Chemical formula	C_29_H_34_FN_5_S	C_30_H_36_FN_5_OS
*M*_r_	503.67	533.7
Crystal system, space group	Triclinic, *P* 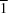	Monoclinic, *P*2_1_/*c*
Temperature (K)	296	293
*a*, *b*, *c* (Å)	10.4173 (5), 10.9849 (5), 12.0002 (6)	11.3074 (7), 12.1576 (8), 20.4976 (13)
α, β, γ (°)	72.769 (2), 84.623 (2), 89.244 (2)	90, 101.328 (2), 90
*V* (Å^3^)	1305.66 (11)	2762.9 (3)
*Z*	2	4
Radiation type	Mo *K*α	Mo *K*α
μ (mm^−1^)	0.16	0.16
Crystal size (mm)	0.45 × 0.33 × 0.09	0.42 × 0.19 × 0.16

Data collection		
Diffractometer	Bruker APEXII CCD	Bruker APEXII CCD
Absorption correction	Multi-scan (*SADABS*; Bruker, 2012 ▸)	Multi-scan (*SADABS*; Bruker, 2012 ▸)
*T*_min_, *T*_max_	0.939, 0.986	0.965, 0.975
No. of measured, independent and observed [*I* > 2σ(*I*)] reflections	49580, 6011, 3533	67287, 6350, 3203
*R*_int_	0.076	0.134
(sin θ/λ)_max_ (Å^−1^)	0.650	0.649

Refinement		
*R*[*F* ^2^ > 2σ(*F* ^2^)], *wR*(*F* ^2^), *S*	0.061, 0.163, 1.03	0.055, 0.133, 1.01
No. of reflections	6007	6347
No. of parameters	325	344
H-atom treatment	H-atom parameters constrained	H-atom parameters constrained
Δρ_max_, Δρ_min_ (e Å^−3^)	0.26, −0.25	0.16, −0.22

Computer programs: *APEX2* and *SAINT* (Bruker, 2012 ▸), *SHELXS2016* (Sheldrick, 2008 ▸), *PLATON* (Spek, 2009 ▸) and *Mercury* (Macrae *et al.*, 2008 ▸), *SHELXL2016* (Sheldrick, 2015 ▸), *WinGX* (Farrugia, 2012 ▸) and *publCIF* (Westrip, 2010 ▸).

## supplementary crystallographic information

### 3-(Adamantan-1-yl)-4-(4-fluorophenyl)-1-[(4-phenylpiperazin-1-yl)methyl]-4,5-dihydro-1H-1,2,4-triazole-5-thione (I) Crystal data

**Table:**

C_29_H_34_FN_5_S	*Z* = 2
*M**_r_* = 503.67	*F*(000) = 536
Triclinic, *P*1	*D*_x_ = 1.281 Mg m^−^^3^
Hall symbol: -P 1	Mo *K*α radiation, λ = 0.71073 Å
*a* = 10.4173 (5) Å	Cell parameters from 9217 reflections
*b* = 10.9849 (5) Å	θ = 2.5–23.7°
*c* = 12.0002 (6) Å	µ = 0.16 mm^−^^1^
α = 72.769 (2)°	*T* = 296 K
β = 84.623 (2)°	Plate, colourless
γ = 89.244 (2)°	0.45 × 0.33 × 0.09 mm
*V* = 1305.66 (11) Å^3^	

### 3-(Adamantan-1-yl)-4-(4-fluorophenyl)-1-[(4-phenylpiperazin-1-yl)methyl]-4,5-dihydro-1H-1,2,4-triazole-5-thione (I) Data collection

**Table:**

Bruker APEXII CCD diffractometer	6011 independent reflections
Radiation source: fine-focus sealed tube	3533 reflections with *I* > 2σ(*I*)
Graphite monochromator	*R*_int_ = 0.076
φ and ω scans	θ_max_ = 27.5°, θ_min_ = 2.5°
Absorption correction: multi-scan (SADABS; Bruker, 2012)	*h* = −13→13
*T*_min_ = 0.939, *T*_max_ = 0.986	*k* = −14→14
49580 measured reflections	*l* = −15→15

### 3-(Adamantan-1-yl)-4-(4-fluorophenyl)-1-[(4-phenylpiperazin-1-yl)methyl]-4,5-dihydro-1H-1,2,4-triazole-5-thione (I) Refinement

**Table:**

Refinement on *F*^2^	Primary atom site location: structure-invariant direct methods
Least-squares matrix: full	Secondary atom site location: difference Fourier map
*R*[*F*^2^ > 2σ(*F*^2^)] = 0.061	Hydrogen site location: inferred from neighbouring sites
*wR*(*F*^2^) = 0.163	H-atom parameters constrained
*S* = 1.03	*w* = 1/[σ^2^(*F*_o_^2^) + (0.065*P*)^2^ + 0.6113*P*] where *P* = (*F*_o_^2^ + 2*F*_c_^2^)/3
6007 reflections	(Δ/σ)_max_ < 0.001
325 parameters	Δρ_max_ = 0.26 e Å^−^^3^
0 restraints	Δρ_min_ = −0.25 e Å^−^^3^
0 constraints	

### 3-(Adamantan-1-yl)-4-(4-fluorophenyl)-1-[(4-phenylpiperazin-1-yl)methyl]-4,5-dihydro-1H-1,2,4-triazole-5-thione (I) Special details

**Table:**

Geometry. All esds (except the esd in the dihedral angle between two l.s. planes) are estimated using the full covariance matrix. The cell esds are taken into account individually in the estimation of esds in distances, angles and torsion angles; correlations between esds in cell parameters are only used when they are defined by crystal symmetry. An approximate (isotropic) treatment of cell esds is used for estimating esds involving l.s. planes.
Refinement. Refinement of F^2^ against ALL reflections. The weighted R-factor wR and goodness of fit S are based on F^2^, conventional R-factors R are based on F, with F set to zero for negative F^2^. The threshold expression of F^2^ > 2sigma(F^2^) is used only for calculating R-factors(gt) etc. and is not relevant to the choice of reflections for refinement. R-factors based on F^2^ are statistically about twice as large as those based on F, and R- factors based on ALL data will be even larger.

### 3-(Adamantan-1-yl)-4-(4-fluorophenyl)-1-[(4-phenylpiperazin-1-yl)methyl]-4,5-dihydro-1H-1,2,4-triazole-5-thione (I) Fractional atomic coordinates and isotropic or equivalent isotropic displacement parameters (Å^2^)

**Table:**

	*x*	*y*	*z*	*U*_iso_*/*U*_eq_	
S1	0.97289 (7)	0.79726 (7)	0.53684 (7)	0.0643 (2)	
F1	0.9953 (2)	0.2584 (2)	0.8886 (2)	0.1173 (9)	
N1	0.74459 (18)	0.67026 (17)	0.62307 (16)	0.0391 (5)	
N2	0.59860 (19)	0.80635 (18)	0.54133 (17)	0.0454 (5)	
N3	0.7211 (2)	0.85604 (18)	0.50475 (17)	0.0472 (5)	
N4	0.7392 (2)	1.01606 (19)	0.30911 (17)	0.0494 (5)	
N5	0.7233 (2)	0.9884 (2)	0.08319 (18)	0.0497 (5)	
C1	0.3802 (2)	0.6521 (3)	0.6157 (2)	0.0528 (7)	
H1A	0.3644	0.7428	0.6098	0.063*	
H1B	0.3933	0.6444	0.5353	0.063*	
C2	0.2634 (3)	0.5709 (3)	0.6825 (3)	0.0592 (7)	
H2A	0.1852	0.6003	0.64	0.071*	
C3	0.2423 (3)	0.5859 (3)	0.8050 (3)	0.0716 (9)	
H3A	0.226	0.6764	0.7997	0.086*	
H3B	0.1661	0.5346	0.8486	0.086*	
C4	0.3614 (3)	0.5414 (3)	0.8693 (2)	0.0660 (8)	
H4A	0.3472	0.5516	0.9496	0.079*	
C5	0.4791 (2)	0.6218 (3)	0.8034 (2)	0.0514 (7)	
H5A	0.5559	0.5932	0.8463	0.062*	
H5B	0.4646	0.7123	0.7986	0.062*	
C6	0.5025 (2)	0.6083 (2)	0.67938 (19)	0.0365 (5)	
C7	0.2865 (3)	0.4317 (3)	0.6911 (3)	0.0669 (8)	
H7A	0.3007	0.4216	0.6117	0.08*	
H7B	0.2101	0.3795	0.7328	0.08*	
C8	0.4048 (3)	0.3872 (3)	0.7577 (3)	0.0606 (8)	
H8A	0.4197	0.2952	0.7647	0.073*	
C9	0.5233 (2)	0.4665 (2)	0.6893 (2)	0.0487 (6)	
H9A	0.5367	0.4557	0.61	0.058*	
H9B	0.6011	0.4362	0.7303	0.058*	
C10	0.3844 (3)	0.4035 (3)	0.8797 (3)	0.0727 (9)	
H10A	0.3093	0.3513	0.9245	0.087*	
H10B	0.4614	0.3744	0.9223	0.087*	
C11	0.6145 (2)	0.6934 (2)	0.61309 (19)	0.0381 (5)	
C12	0.8141 (2)	0.7769 (2)	0.5529 (2)	0.0442 (6)	
C13	0.8086 (2)	0.5622 (2)	0.6933 (2)	0.0393 (5)	
C14	0.8596 (2)	0.4721 (2)	0.6427 (2)	0.0505 (6)	
H14A	0.8511	0.4817	0.5624	0.061*	
C15	0.9223 (3)	0.3690 (3)	0.7086 (3)	0.0648 (8)	
H15A	0.9572	0.3058	0.6753	0.078*	
C16	0.9332 (3)	0.3597 (3)	0.8221 (3)	0.0700 (9)	
C17	0.8867 (3)	0.4476 (3)	0.8746 (2)	0.0679 (9)	
H17A	0.897	0.4375	0.9546	0.081*	
C18	0.8236 (2)	0.5524 (3)	0.8079 (2)	0.0520 (7)	
H18A	0.7913	0.6164	0.8412	0.062*	
C19	0.7401 (3)	0.9915 (2)	0.4322 (2)	0.0548 (7)	
H19A	0.6716	1.0425	0.459	0.066*	
H19B	0.8236	1.0223	0.4476	0.066*	
C20	0.6161 (3)	1.0001 (3)	0.2677 (2)	0.0530 (7)	
H20A	0.5484	1.0444	0.3039	0.064*	
H20B	0.5925	0.9084	0.2908	0.064*	
C21	0.6245 (3)	1.0548 (3)	0.1353 (2)	0.0530 (7)	
H21A	0.5403	1.0445	0.1069	0.064*	
H21B	0.6462	1.1469	0.1121	0.064*	
C23	0.8397 (3)	0.9514 (3)	0.2571 (2)	0.0574 (7)	
H23A	0.8203	0.8588	0.2814	0.069*	
H23B	0.9236	0.9644	0.2848	0.069*	
C22	0.8475 (3)	1.0037 (3)	0.1250 (2)	0.0585 (7)	
H22A	0.8722	1.0951	0.1006	0.07*	
H22B	0.9145	0.958	0.0897	0.07*	
C24	0.7295 (3)	1.0128 (2)	−0.0403 (2)	0.0502 (6)	
C25	0.8004 (3)	0.9305 (3)	−0.0903 (3)	0.0678 (8)	
H25A	0.8423	0.8599	−0.0417	0.081*	
C26	0.8102 (4)	0.9509 (4)	−0.2097 (3)	0.0820 (10)	
H26A	0.8584	0.8938	−0.2426	0.098*	
C27	0.7515 (4)	1.0521 (4)	−0.2813 (3)	0.0837 (11)	
H27A	0.7599	1.066	−0.3637	0.1*	
C28	0.6804 (3)	1.1335 (4)	−0.2340 (3)	0.0782 (10)	
H28A	0.6387	1.2036	−0.2835	0.094*	
C29	0.6692 (3)	1.1139 (3)	−0.1138 (3)	0.0633 (8)	
H29A	0.6195	1.1706	−0.0816	0.076*	

### 3-(Adamantan-1-yl)-4-(4-fluorophenyl)-1-[(4-phenylpiperazin-1-yl)methyl]-4,5-dihydro-1H-1,2,4-triazole-5-thione (I) Atomic displacement parameters (Å^2^)

**Table:**

	*U*^11^	*U*^22^	*U*^33^	*U*^12^	*U*^13^	*U*^23^
S1	0.0496 (4)	0.0660 (5)	0.0691 (5)	−0.0162 (3)	0.0024 (3)	−0.0092 (4)
F1	0.0819 (14)	0.1034 (16)	0.1216 (18)	0.0377 (12)	−0.0074 (12)	0.0334 (13)
N1	0.0401 (11)	0.0368 (11)	0.0371 (11)	−0.0016 (8)	0.0025 (8)	−0.0076 (8)
N2	0.0497 (12)	0.0399 (11)	0.0410 (11)	0.0049 (9)	0.0022 (9)	−0.0058 (9)
N3	0.0543 (13)	0.0385 (11)	0.0415 (12)	−0.0042 (10)	0.0042 (9)	−0.0030 (9)
N4	0.0572 (13)	0.0475 (12)	0.0411 (12)	−0.0079 (10)	−0.0001 (10)	−0.0107 (9)
N5	0.0502 (13)	0.0550 (13)	0.0439 (12)	0.0054 (10)	−0.0022 (9)	−0.0155 (10)
C1	0.0481 (15)	0.0545 (16)	0.0541 (16)	0.0109 (12)	−0.0079 (12)	−0.0130 (13)
C2	0.0377 (14)	0.0703 (19)	0.074 (2)	0.0088 (13)	−0.0132 (13)	−0.0255 (15)
C3	0.0447 (16)	0.090 (2)	0.085 (2)	−0.0052 (15)	0.0155 (15)	−0.0409 (18)
C4	0.0536 (17)	0.098 (2)	0.0467 (16)	−0.0167 (16)	0.0137 (13)	−0.0262 (16)
C5	0.0447 (14)	0.0666 (17)	0.0468 (15)	−0.0077 (12)	0.0044 (11)	−0.0248 (13)
C6	0.0380 (12)	0.0370 (12)	0.0341 (12)	0.0051 (10)	0.0005 (9)	−0.0114 (10)
C7	0.0460 (16)	0.075 (2)	0.089 (2)	−0.0099 (14)	−0.0048 (15)	−0.0379 (17)
C8	0.0475 (15)	0.0402 (15)	0.093 (2)	−0.0066 (12)	−0.0009 (14)	−0.0186 (14)
C9	0.0415 (14)	0.0434 (14)	0.0637 (17)	0.0020 (11)	−0.0014 (12)	−0.0206 (12)
C10	0.0544 (18)	0.081 (2)	0.064 (2)	−0.0212 (16)	0.0055 (14)	0.0049 (16)
C11	0.0438 (13)	0.0378 (13)	0.0320 (12)	0.0050 (10)	0.0003 (10)	−0.0107 (10)
C12	0.0507 (15)	0.0422 (14)	0.0381 (13)	−0.0036 (11)	0.0031 (11)	−0.0114 (11)
C13	0.0324 (12)	0.0402 (13)	0.0413 (14)	−0.0017 (10)	0.0008 (10)	−0.0073 (10)
C14	0.0423 (14)	0.0535 (16)	0.0567 (16)	0.0025 (12)	−0.0017 (12)	−0.0185 (13)
C15	0.0451 (16)	0.0540 (18)	0.092 (2)	0.0079 (13)	−0.0001 (15)	−0.0185 (16)
C16	0.0418 (16)	0.066 (2)	0.079 (2)	0.0101 (14)	0.0003 (15)	0.0128 (17)
C17	0.0473 (16)	0.099 (2)	0.0415 (16)	0.0024 (16)	−0.0036 (12)	0.0037 (16)
C18	0.0403 (14)	0.0688 (18)	0.0453 (15)	−0.0011 (12)	0.0010 (11)	−0.0160 (13)
C19	0.0785 (19)	0.0372 (14)	0.0445 (15)	−0.0067 (13)	−0.0011 (13)	−0.0069 (11)
C20	0.0562 (16)	0.0494 (15)	0.0514 (16)	0.0017 (12)	0.0045 (12)	−0.0148 (12)
C21	0.0516 (15)	0.0584 (16)	0.0490 (16)	0.0068 (13)	−0.0034 (12)	−0.0164 (13)
C23	0.0482 (15)	0.0715 (19)	0.0477 (16)	−0.0004 (13)	−0.0034 (12)	−0.0105 (13)
C22	0.0480 (16)	0.076 (2)	0.0475 (16)	0.0016 (14)	−0.0001 (12)	−0.0136 (14)
C24	0.0522 (15)	0.0520 (16)	0.0479 (15)	−0.0042 (12)	−0.0024 (12)	−0.0176 (12)
C25	0.076 (2)	0.069 (2)	0.063 (2)	0.0030 (16)	−0.0014 (16)	−0.0289 (16)
C26	0.084 (2)	0.103 (3)	0.073 (2)	−0.006 (2)	0.0066 (19)	−0.051 (2)
C27	0.086 (2)	0.117 (3)	0.0518 (19)	−0.022 (2)	−0.0013 (18)	−0.031 (2)
C28	0.079 (2)	0.094 (3)	0.056 (2)	−0.0078 (19)	−0.0103 (17)	−0.0124 (18)
C29	0.0690 (19)	0.0678 (19)	0.0543 (18)	0.0011 (15)	−0.0054 (14)	−0.0200 (15)

### 3-(Adamantan-1-yl)-4-(4-fluorophenyl)-1-[(4-phenylpiperazin-1-yl)methyl]-4,5-dihydro-1H-1,2,4-triazole-5-thione (I) Geometric parameters (Å, º)

**Table:**

S1—C12	1.658 (3)	C8—H8A	1
F1—C16	1.358 (3)	C9—H9A	0.99
N1—C11	1.385 (3)	C9—H9B	0.99
N1—C12	1.389 (3)	C10—H10A	0.99
N1—C13	1.433 (3)	C10—H10B	0.99
N2—C11	1.303 (3)	C13—C18	1.371 (3)
N2—N3	1.375 (3)	C13—C14	1.383 (3)
N3—C12	1.346 (3)	C14—C15	1.373 (4)
N3—C19	1.490 (3)	C14—H14A	0.95
N4—C19	1.422 (3)	C15—C16	1.351 (5)
N4—C20	1.449 (3)	C15—H15A	0.95
N4—C23	1.452 (3)	C16—C17	1.361 (5)
N5—C24	1.421 (3)	C17—C18	1.389 (4)
N5—C21	1.453 (3)	C17—H17A	0.95
N5—C22	1.459 (3)	C18—H18A	0.95
C1—C2	1.530 (4)	C19—H19A	0.99
C1—C6	1.545 (3)	C19—H19B	0.99
C1—H1A	0.99	C20—C21	1.518 (3)
C1—H1B	0.99	C20—H20A	0.99
C2—C7	1.519 (4)	C20—H20B	0.99
C2—C3	1.523 (4)	C21—H21A	0.99
C2—H2A	1	C21—H21B	0.99
C3—C4	1.521 (4)	C23—C22	1.513 (4)
C3—H3A	0.99	C23—H23A	0.99
C3—H3B	0.99	C23—H23B	0.99
C4—C10	1.501 (4)	C22—H22A	0.99
C4—C5	1.529 (4)	C22—H22B	0.99
C4—H4A	1	C24—C29	1.383 (4)
C5—C6	1.536 (3)	C24—C25	1.394 (4)
C5—H5A	0.99	C25—C26	1.377 (4)
C5—H5B	0.99	C25—H25A	0.95
C6—C11	1.511 (3)	C26—C27	1.365 (5)
C6—C9	1.540 (3)	C26—H26A	0.95
C7—C8	1.528 (4)	C27—C28	1.370 (5)
C7—H7A	0.99	C27—H27A	0.95
C7—H7B	0.99	C28—C29	1.387 (4)
C8—C10	1.522 (4)	C28—H28A	0.95
C8—C9	1.539 (4)	C29—H29A	0.95
			
C11—N1—C12	108.39 (19)	N2—C11—N1	110.2 (2)
C11—N1—C13	130.57 (18)	N2—C11—C6	122.5 (2)
C12—N1—C13	121.01 (19)	N1—C11—C6	127.21 (19)
C11—N2—N3	105.12 (19)	N3—C12—N1	102.9 (2)
C12—N3—N2	113.44 (18)	N3—C12—S1	130.23 (18)
C12—N3—C19	125.9 (2)	N1—C12—S1	126.90 (19)
N2—N3—C19	120.1 (2)	C18—C13—C14	120.9 (2)
C19—N4—C20	116.2 (2)	C18—C13—N1	119.8 (2)
C19—N4—C23	114.0 (2)	C14—C13—N1	119.3 (2)
C20—N4—C23	110.5 (2)	C15—C14—C13	119.9 (3)
C24—N5—C21	116.6 (2)	C15—C14—H14A	120.1
C24—N5—C22	113.3 (2)	C13—C14—H14A	120.1
C21—N5—C22	109.8 (2)	C16—C15—C14	118.1 (3)
C2—C1—C6	110.4 (2)	C16—C15—H15A	120.9
C2—C1—H1A	109.6	C14—C15—H15A	120.9
C6—C1—H1A	109.6	C15—C16—F1	118.9 (3)
C2—C1—H1B	109.6	C15—C16—C17	123.8 (3)
C6—C1—H1B	109.6	F1—C16—C17	117.3 (3)
H1A—C1—H1B	108.1	C16—C17—C18	118.2 (3)
C7—C2—C3	109.6 (3)	C16—C17—H17A	120.9
C7—C2—C1	109.9 (2)	C18—C17—H17A	120.9
C3—C2—C1	109.2 (2)	C13—C18—C17	119.1 (3)
C7—C2—H2A	109.4	C13—C18—H18A	120.5
C3—C2—H2A	109.4	C17—C18—H18A	120.5
C1—C2—H2A	109.4	N4—C19—N3	116.3 (2)
C4—C3—C2	109.3 (2)	N4—C19—H19A	108.2
C4—C3—H3A	109.8	N3—C19—H19A	108.2
C2—C3—H3A	109.8	N4—C19—H19B	108.2
C4—C3—H3B	109.8	N3—C19—H19B	108.2
C2—C3—H3B	109.8	H19A—C19—H19B	107.4
H3A—C3—H3B	108.3	N4—C20—C21	109.7 (2)
C10—C4—C3	109.9 (3)	N4—C20—H20A	109.7
C10—C4—C5	109.9 (2)	C21—C20—H20A	109.7
C3—C4—C5	110.1 (3)	N4—C20—H20B	109.7
C10—C4—H4A	109	C21—C20—H20B	109.7
C3—C4—H4A	109	H20A—C20—H20B	108.2
C5—C4—H4A	109	N5—C21—C20	109.2 (2)
C4—C5—C6	110.0 (2)	N5—C21—H21A	109.8
C4—C5—H5A	109.7	C20—C21—H21A	109.8
C6—C5—H5A	109.7	N5—C21—H21B	109.8
C4—C5—H5B	109.7	C20—C21—H21B	109.8
C6—C5—H5B	109.7	H21A—C21—H21B	108.3
H5A—C5—H5B	108.2	N4—C23—C22	109.7 (2)
C11—C6—C5	109.46 (19)	N4—C23—H23A	109.7
C11—C6—C9	113.41 (18)	C22—C23—H23A	109.7
C5—C6—C9	108.6 (2)	N4—C23—H23B	109.7
C11—C6—C1	108.72 (18)	C22—C23—H23B	109.7
C5—C6—C1	108.15 (19)	H23A—C23—H23B	108.2
C9—C6—C1	108.4 (2)	N5—C22—C23	110.3 (2)
C2—C7—C8	108.9 (2)	N5—C22—H22A	109.6
C2—C7—H7A	109.9	C23—C22—H22A	109.6
C8—C7—H7A	109.9	N5—C22—H22B	109.6
C2—C7—H7B	109.9	C23—C22—H22B	109.6
C8—C7—H7B	109.9	H22A—C22—H22B	108.1
H7A—C7—H7B	108.3	C29—C24—C25	118.1 (3)
C10—C8—C7	110.3 (2)	C29—C24—N5	123.6 (2)
C10—C8—C9	109.7 (2)	C25—C24—N5	118.3 (2)
C7—C8—C9	109.1 (2)	C26—C25—C24	120.4 (3)
C10—C8—H8A	109.2	C26—C25—H25A	119.8
C7—C8—H8A	109.2	C24—C25—H25A	119.8
C9—C8—H8A	109.2	C27—C26—C25	120.8 (3)
C8—C9—C6	109.8 (2)	C27—C26—H26A	119.6
C8—C9—H9A	109.7	C25—C26—H26A	119.6
C6—C9—H9A	109.7	C26—C27—C28	119.8 (3)
C8—C9—H9B	109.7	C26—C27—H27A	120.1
C6—C9—H9B	109.7	C28—C27—H27A	120.1
H9A—C9—H9B	108.2	C27—C28—C29	120.1 (3)
C4—C10—C8	109.3 (2)	C27—C28—H28A	120
C4—C10—H10A	109.8	C29—C28—H28A	120
C8—C10—H10A	109.8	C24—C29—C28	120.8 (3)
C4—C10—H10B	109.8	C24—C29—H29A	119.6
C8—C10—H10B	109.8	C28—C29—H29A	119.6
H10A—C10—H10B	108.3		
			
C11—N2—N3—C12	−0.5 (3)	C11—N1—C12—N3	−0.6 (2)
C11—N2—N3—C19	−172.6 (2)	C13—N1—C12—N3	−178.6 (2)
C6—C1—C2—C7	−59.7 (3)	C11—N1—C12—S1	178.75 (18)
C6—C1—C2—C3	60.6 (3)	C13—N1—C12—S1	0.8 (3)
C7—C2—C3—C4	60.3 (3)	C11—N1—C13—C18	−83.5 (3)
C1—C2—C3—C4	−60.2 (3)	C12—N1—C13—C18	94.0 (3)
C2—C3—C4—C10	−60.7 (3)	C11—N1—C13—C14	99.5 (3)
C2—C3—C4—C5	60.4 (3)	C12—N1—C13—C14	−83.1 (3)
C10—C4—C5—C6	60.9 (3)	C18—C13—C14—C15	2.3 (4)
C3—C4—C5—C6	−60.2 (3)	N1—C13—C14—C15	179.3 (2)
C4—C5—C6—C11	177.0 (2)	C13—C14—C15—C16	−0.6 (4)
C4—C5—C6—C9	−58.7 (3)	C14—C15—C16—F1	−179.9 (3)
C4—C5—C6—C1	58.7 (3)	C14—C15—C16—C17	−0.8 (5)
C2—C1—C6—C11	−178.1 (2)	C15—C16—C17—C18	0.5 (5)
C2—C1—C6—C5	−59.4 (3)	F1—C16—C17—C18	179.6 (2)
C2—C1—C6—C9	58.2 (3)	C14—C13—C18—C17	−2.5 (4)
C3—C2—C7—C8	−59.2 (3)	N1—C13—C18—C17	−179.5 (2)
C1—C2—C7—C8	60.8 (3)	C16—C17—C18—C13	1.2 (4)
C2—C7—C8—C10	59.0 (3)	C20—N4—C19—N3	69.5 (3)
C2—C7—C8—C9	−61.5 (3)	C23—N4—C19—N3	−60.7 (3)
C10—C8—C9—C6	−59.5 (3)	C12—N3—C19—N4	102.6 (3)
C7—C8—C9—C6	61.4 (3)	N2—N3—C19—N4	−86.3 (3)
C11—C6—C9—C8	−179.9 (2)	C19—N4—C20—C21	168.6 (2)
C5—C6—C9—C8	58.2 (3)	C23—N4—C20—C21	−59.5 (3)
C1—C6—C9—C8	−59.1 (3)	C24—N5—C21—C20	169.9 (2)
C3—C4—C10—C8	60.1 (3)	C22—N5—C21—C20	−59.5 (3)
C5—C4—C10—C8	−61.2 (3)	N4—C20—C21—N5	59.9 (3)
C7—C8—C10—C4	−59.6 (3)	C19—N4—C23—C22	−168.7 (2)
C9—C8—C10—C4	60.6 (3)	C20—N4—C23—C22	58.3 (3)
N3—N2—C11—N1	0.1 (2)	C24—N5—C22—C23	−168.7 (2)
N3—N2—C11—C6	176.33 (19)	C21—N5—C22—C23	59.0 (3)
C12—N1—C11—N2	0.3 (3)	N4—C23—C22—N5	−57.8 (3)
C13—N1—C11—N2	178.0 (2)	C21—N5—C24—C29	15.0 (4)
C12—N1—C11—C6	−175.7 (2)	C22—N5—C24—C29	−113.9 (3)
C13—N1—C11—C6	2.0 (4)	C21—N5—C24—C25	−165.1 (3)
C5—C6—C11—N2	−97.1 (3)	C22—N5—C24—C25	66.0 (3)
C9—C6—C11—N2	141.4 (2)	C29—C24—C25—C26	0.5 (4)
C1—C6—C11—N2	20.8 (3)	N5—C24—C25—C26	−179.4 (3)
C5—C6—C11—N1	78.4 (3)	C24—C25—C26—C27	0.4 (5)
C9—C6—C11—N1	−43.0 (3)	C25—C26—C27—C28	−1.0 (5)
C1—C6—C11—N1	−163.6 (2)	C26—C27—C28—C29	0.7 (5)
N2—N3—C12—N1	0.7 (3)	C25—C24—C29—C28	−0.8 (4)
C19—N3—C12—N1	172.3 (2)	N5—C24—C29—C28	179.1 (3)
N2—N3—C12—S1	−178.63 (18)	C27—C28—C29—C24	0.2 (5)
C19—N3—C12—S1	−7.1 (4)		

### 3-(Adamantan-1-yl)-4-(4-fluorophenyl)-1-[(4-phenylpiperazin-1-yl)methyl]-4,5-dihydro-1H-1,2,4-triazole-5-thione (I) Hydrogen-bond geometry (Å, º)

**Table:**

*D*—H···*A*	*D*—H	H···*A*	*D*···*A*	*D*—H···*A*
C22—H22*B*···F1^i^	0.99	2.49	3.332 (4)	142

Symmetry code: (i) −*x*+2, −*y*+1, −*z*+1.

### 3-(Adamantan-1-yl)-4-(4-fluorophenyl)-1-{[4-(2-methoxyphenyl)piperazin-1-yl]methyl}-4,5-dihydro-1H-1,2,4-triazole-5-thione (II) Crystal data

**Table:**

C_30_H_36_FN_5_OS	*F*(000) = 1136
*M**_r_* = 533.7	*D*_x_ = 1.283 Mg m^−^^3^
Monoclinic, *P*2_1_/*c*	Mo *K*α radiation, λ = 0.71073 Å
Hall symbol: -P 2ybc	Cell parameters from 8790 reflections
*a* = 11.3074 (7) Å	θ = 2.5–22.8°
*b* = 12.1576 (8) Å	µ = 0.16 mm^−^^1^
*c* = 20.4976 (13) Å	*T* = 293 K
β = 101.328 (2)°	Block, colourless
*V* = 2762.9 (3) Å^3^	0.42 × 0.19 × 0.16 mm
*Z* = 4	

### 3-(Adamantan-1-yl)-4-(4-fluorophenyl)-1-{[4-(2-methoxyphenyl)piperazin-1-yl]methyl}-4,5-dihydro-1H-1,2,4-triazole-5-thione (II) Data collection

**Table:**

Bruker APEXII CCD diffractometer	6350 independent reflections
Radiation source: fine-focus sealed tube	3203 reflections with *I* > 2σ(*I*)
Graphite monochromator	*R*_int_ = 0.134
φ and ω scans	θ_max_ = 27.5°, θ_min_ = 2.5°
Absorption correction: multi-scan (SADABS; Bruker, 2012)	*h* = −14→14
*T*_min_ = 0.965, *T*_max_ = 0.975	*k* = −15→15
67287 measured reflections	*l* = −26→26

### 3-(Adamantan-1-yl)-4-(4-fluorophenyl)-1-{[4-(2-methoxyphenyl)piperazin-1-yl]methyl}-4,5-dihydro-1H-1,2,4-triazole-5-thione (II) Refinement

**Table:**

Refinement on *F*^2^	Primary atom site location: structure-invariant direct methods
Least-squares matrix: full	Secondary atom site location: difference Fourier map
*R*[*F*^2^ > 2σ(*F*^2^)] = 0.055	Hydrogen site location: inferred from neighbouring sites
*wR*(*F*^2^) = 0.133	H-atom parameters constrained
*S* = 1.01	*w* = 1/[σ^2^(*F*_o_^2^) + (0.0452*P*)^2^ + 0.8222*P*] where *P* = (*F*_o_^2^ + 2*F*_c_^2^)/3
6347 reflections	(Δ/σ)_max_ < 0.001
344 parameters	Δρ_max_ = 0.16 e Å^−^^3^
0 restraints	Δρ_min_ = −0.22 e Å^−^^3^
0 constraints	

### 3-(Adamantan-1-yl)-4-(4-fluorophenyl)-1-{[4-(2-methoxyphenyl)piperazin-1-yl]methyl}-4,5-dihydro-1H-1,2,4-triazole-5-thione (II) Special details

**Table:**

Geometry. All esds (except the esd in the dihedral angle between two l.s. planes) are estimated using the full covariance matrix. The cell esds are taken into account individually in the estimation of esds in distances, angles and torsion angles; correlations between esds in cell parameters are only used when they are defined by crystal symmetry. An approximate (isotropic) treatment of cell esds is used for estimating esds involving l.s. planes.
Refinement. Refinement of F^2^ against ALL reflections. The weighted R-factor wR and goodness of fit S are based on F^2^, conventional R-factors R are based on F, with F set to zero for negative F^2^. The threshold expression of F^2^ > 2sigma(F^2^) is used only for calculating R-factors(gt) etc. and is not relevant to the choice of reflections for refinement. R-factors based on F^2^ are statistically about twice as large as those based on F, and R- factors based on ALL data will be even larger.

### 3-(Adamantan-1-yl)-4-(4-fluorophenyl)-1-{[4-(2-methoxyphenyl)piperazin-1-yl]methyl}-4,5-dihydro-1H-1,2,4-triazole-5-thione (II) Fractional atomic coordinates and isotropic or equivalent isotropic displacement parameters (Å^2^)

**Table:**

	*x*	*y*	*z*	*U*_iso_*/*U*_eq_	
S1	0.24167 (6)	0.32667 (6)	0.43345 (3)	0.0608 (2)	
F1	0.3856 (2)	0.79567 (15)	0.45842 (10)	0.1199 (8)	
O1	0.34615 (16)	−0.06806 (14)	0.77358 (8)	0.0618 (5)	
N1	0.19559 (16)	0.42142 (14)	0.54744 (10)	0.0428 (5)	
N3	0.13241 (17)	0.25598 (15)	0.53226 (9)	0.0469 (5)	
N2	0.10390 (17)	0.28893 (15)	0.59193 (10)	0.0481 (5)	
N4	0.18293 (18)	0.05911 (15)	0.54290 (9)	0.0476 (5)	
N5	0.35533 (17)	−0.04464 (14)	0.64472 (9)	0.0439 (5)	
C1	0.0665 (2)	0.5658 (2)	0.64261 (13)	0.0583 (7)	
H1A	0.1087	0.6099	0.615	0.07*	
H1B	−0.0137	0.5502	0.6174	0.07*	
C2	0.0572 (3)	0.6293 (2)	0.70571 (14)	0.0645 (8)	
H2A	0.014	0.6984	0.6932	0.077*	
C3	−0.0100 (3)	0.5635 (2)	0.74851 (16)	0.0755 (9)	
H3A	−0.0907	0.5476	0.7242	0.091*	
H3B	−0.0167	0.6053	0.7879	0.091*	
C4	0.0574 (3)	0.4566 (2)	0.76852 (15)	0.0750 (9)	
H4A	0.0135	0.4131	0.7962	0.09*	
C5	0.0666 (3)	0.3909 (2)	0.70539 (14)	0.0680 (8)	
H5A	0.1084	0.3221	0.7179	0.082*	
H5B	−0.0137	0.3739	0.6808	0.082*	
C6	0.13488 (19)	0.45742 (17)	0.66124 (11)	0.0412 (6)	
C7	0.1822 (3)	0.6551 (2)	0.74427 (15)	0.0741 (9)	
H7A	0.2254	0.6987	0.717	0.089*	
H7B	0.1765	0.6975	0.7836	0.089*	
C8	0.2498 (2)	0.5495 (2)	0.76445 (14)	0.0688 (8)	
H8A	0.3305	0.5668	0.7897	0.083*	
C9	0.2610 (2)	0.4844 (2)	0.70174 (13)	0.0596 (7)	
H9A	0.3051	0.4168	0.7143	0.072*	
H9B	0.3054	0.5274	0.6748	0.072*	
C10	0.1828 (3)	0.4810 (3)	0.80713 (14)	0.0807 (9)	
H10A	0.2258	0.4127	0.8194	0.097*	
H10B	0.1779	0.5207	0.8476	0.097*	
C11	0.14343 (19)	0.39012 (18)	0.60057 (11)	0.0413 (6)	
C12	0.1899 (2)	0.33355 (19)	0.50378 (12)	0.0455 (6)	
C13	0.2440 (2)	0.52480 (18)	0.53089 (11)	0.0429 (6)	
C14	0.3666 (2)	0.5415 (2)	0.54450 (13)	0.0610 (7)	
H14A	0.4173	0.4902	0.5695	0.073*	
C15	0.4142 (3)	0.6341 (3)	0.52107 (16)	0.0785 (10)	
H15A	0.4969	0.6466	0.53	0.094*	
C16	0.3373 (4)	0.7071 (2)	0.48460 (16)	0.0740 (9)	
C17	0.2163 (3)	0.6944 (2)	0.47141 (14)	0.0666 (8)	
H17A	0.1662	0.7472	0.4474	0.08*	
C18	0.1692 (2)	0.60109 (19)	0.49462 (12)	0.0514 (6)	
H18A	0.0862	0.5897	0.4857	0.062*	
C19	0.1079 (2)	0.14220 (19)	0.50729 (12)	0.0550 (7)	
H19A	0.0246	0.1246	0.5083	0.066*	
H19B	0.1165	0.1399	0.4612	0.066*	
C20	0.3118 (2)	0.0819 (2)	0.55064 (12)	0.0541 (7)	
H20A	0.3322	0.146	0.5788	0.065*	
H20B	0.3305	0.0983	0.5075	0.065*	
C21	0.3869 (2)	−0.0149 (2)	0.58108 (12)	0.0560 (7)	
H21A	0.3728	−0.0772	0.551	0.067*	
H21B	0.4719	0.0039	0.588	0.067*	
C22	0.2259 (2)	−0.06913 (19)	0.63489 (12)	0.0505 (6)	
H22A	0.2053	−0.0894	0.677	0.061*	
H22B	0.2066	−0.1307	0.6046	0.061*	
C23	0.1536 (2)	0.03040 (19)	0.60675 (12)	0.0483 (6)	
H23A	0.0681	0.0145	0.6011	0.058*	
H23B	0.1721	0.0918	0.6373	0.058*	
C24	0.4291 (2)	−0.12587 (18)	0.68294 (12)	0.0453 (6)	
C25	0.5089 (2)	−0.1917 (2)	0.65777 (13)	0.0592 (7)	
H25A	0.5185	−0.1814	0.6142	0.071*	
C26	0.5751 (3)	−0.2730 (2)	0.69618 (16)	0.0722 (8)	
H26A	0.6289	−0.316	0.6783	0.087*	
C27	0.5616 (2)	−0.2903 (2)	0.76022 (16)	0.0686 (8)	
H27A	0.6031	−0.3471	0.7852	0.082*	
C28	0.4857 (2)	−0.2226 (2)	0.78764 (13)	0.0591 (7)	
H28A	0.4781	−0.2326	0.8316	0.071*	
C29	0.4211 (2)	−0.14002 (19)	0.74968 (13)	0.0482 (6)	
C30	0.3295 (3)	−0.0813 (2)	0.83902 (13)	0.0715 (8)	
H30A	0.2762	−0.0251	0.8491	0.107*	
H30B	0.4059	−0.0755	0.8691	0.107*	
H30C	0.295	−0.1523	0.8437	0.107*	

### 3-(Adamantan-1-yl)-4-(4-fluorophenyl)-1-{[4-(2-methoxyphenyl)piperazin-1-yl]methyl}-4,5-dihydro-1H-1,2,4-triazole-5-thione (II) Atomic displacement parameters (Å^2^)

**Table:**

	*U*^11^	*U*^22^	*U*^33^	*U*^12^	*U*^13^	*U*^23^
S1	0.0707 (5)	0.0622 (4)	0.0515 (4)	0.0101 (4)	0.0172 (3)	−0.0018 (3)
F1	0.190 (2)	0.0819 (13)	0.1131 (15)	−0.0717 (13)	0.0917 (15)	−0.0292 (11)
O1	0.0783 (13)	0.0624 (12)	0.0492 (11)	0.0115 (10)	0.0236 (9)	0.0040 (9)
N1	0.0404 (11)	0.0354 (11)	0.0527 (12)	0.0050 (9)	0.0089 (9)	−0.0016 (10)
N3	0.0552 (12)	0.0383 (11)	0.0467 (12)	0.0066 (10)	0.0083 (10)	−0.0046 (10)
N2	0.0510 (12)	0.0386 (12)	0.0548 (13)	0.0025 (9)	0.0107 (10)	−0.0056 (10)
N4	0.0623 (14)	0.0379 (11)	0.0429 (12)	0.0048 (10)	0.0111 (10)	−0.0022 (9)
N5	0.0504 (12)	0.0391 (11)	0.0444 (12)	0.0031 (9)	0.0144 (9)	0.0027 (9)
C1	0.0616 (17)	0.0520 (16)	0.0596 (17)	0.0177 (13)	0.0078 (13)	−0.0084 (13)
C2	0.078 (2)	0.0475 (15)	0.0713 (19)	0.0150 (14)	0.0220 (17)	−0.0104 (14)
C3	0.0684 (19)	0.074 (2)	0.093 (2)	−0.0041 (16)	0.0361 (17)	−0.0302 (17)
C4	0.104 (2)	0.0573 (18)	0.080 (2)	−0.0273 (17)	0.059 (2)	−0.0141 (16)
C5	0.087 (2)	0.0501 (16)	0.077 (2)	−0.0167 (14)	0.0416 (17)	−0.0140 (14)
C6	0.0379 (13)	0.0357 (13)	0.0507 (14)	0.0002 (10)	0.0109 (11)	−0.0047 (11)
C7	0.100 (2)	0.0554 (18)	0.073 (2)	−0.0244 (17)	0.0324 (18)	−0.0197 (15)
C8	0.0542 (17)	0.088 (2)	0.0603 (18)	−0.0096 (16)	0.0028 (14)	−0.0189 (16)
C9	0.0476 (15)	0.0727 (18)	0.0578 (17)	0.0027 (13)	0.0086 (13)	−0.0064 (14)
C10	0.115 (3)	0.075 (2)	0.0538 (18)	0.0137 (19)	0.0211 (19)	0.0017 (16)
C11	0.0348 (12)	0.0383 (14)	0.0496 (15)	0.0037 (10)	0.0055 (11)	−0.0008 (11)
C12	0.0408 (13)	0.0425 (14)	0.0509 (15)	0.0101 (11)	0.0033 (12)	0.0030 (12)
C13	0.0398 (14)	0.0430 (14)	0.0469 (14)	−0.0009 (11)	0.0110 (11)	−0.0053 (11)
C14	0.0442 (16)	0.0736 (19)	0.0639 (18)	−0.0019 (14)	0.0076 (13)	−0.0123 (15)
C15	0.062 (2)	0.101 (3)	0.078 (2)	−0.037 (2)	0.0290 (18)	−0.036 (2)
C16	0.108 (3)	0.0583 (19)	0.068 (2)	−0.038 (2)	0.049 (2)	−0.0253 (16)
C17	0.095 (2)	0.0471 (17)	0.0617 (19)	−0.0013 (16)	0.0260 (17)	0.0011 (14)
C18	0.0515 (15)	0.0464 (15)	0.0565 (16)	0.0025 (12)	0.0112 (13)	−0.0004 (13)
C19	0.0658 (17)	0.0420 (15)	0.0519 (16)	0.0003 (12)	−0.0017 (13)	−0.0088 (12)
C20	0.0679 (18)	0.0503 (15)	0.0493 (16)	0.0090 (13)	0.0246 (13)	0.0092 (12)
C21	0.0662 (17)	0.0518 (16)	0.0546 (16)	0.0119 (13)	0.0231 (14)	0.0034 (13)
C22	0.0526 (16)	0.0437 (14)	0.0548 (16)	−0.0053 (12)	0.0095 (12)	0.0005 (12)
C23	0.0492 (14)	0.0427 (14)	0.0539 (16)	−0.0028 (11)	0.0123 (12)	−0.0019 (12)
C24	0.0510 (14)	0.0332 (12)	0.0514 (16)	−0.0025 (11)	0.0093 (12)	−0.0007 (11)
C25	0.0704 (18)	0.0520 (16)	0.0562 (17)	0.0172 (14)	0.0147 (14)	0.0009 (13)
C26	0.077 (2)	0.0632 (19)	0.078 (2)	0.0246 (16)	0.0208 (17)	0.0007 (17)
C27	0.0634 (18)	0.0545 (17)	0.083 (2)	0.0115 (14)	0.0028 (16)	0.0106 (16)
C28	0.0621 (17)	0.0562 (16)	0.0572 (17)	−0.0057 (14)	0.0073 (14)	0.0131 (14)
C29	0.0474 (15)	0.0418 (14)	0.0559 (17)	−0.0045 (11)	0.0113 (13)	0.0020 (12)
C30	0.083 (2)	0.084 (2)	0.0484 (17)	−0.0038 (16)	0.0137 (15)	−0.0073 (15)

### 3-(Adamantan-1-yl)-4-(4-fluorophenyl)-1-{[4-(2-methoxyphenyl)piperazin-1-yl]methyl}-4,5-dihydro-1H-1,2,4-triazole-5-thione (II) Geometric parameters (Å, º)

**Table:**

S1—C12	1.661 (2)	C8—H8A	0.98
F1—C16	1.364 (3)	C9—H9A	0.97
O1—C29	1.374 (3)	C9—H9B	0.97
O1—C30	1.400 (3)	C10—H10A	0.97
N1—C12	1.387 (3)	C10—H10B	0.97
N1—C11	1.390 (3)	C13—C18	1.372 (3)
N1—C13	1.438 (3)	C13—C14	1.374 (3)
N3—C12	1.342 (3)	C14—C15	1.375 (4)
N3—N2	1.384 (3)	C14—H14A	0.93
N3—C19	1.482 (3)	C15—C16	1.358 (4)
N2—C11	1.309 (3)	C15—H15A	0.93
N4—C19	1.425 (3)	C16—C17	1.351 (4)
N4—C23	1.454 (3)	C17—C18	1.377 (3)
N4—C20	1.461 (3)	C17—H17A	0.93
N5—C24	1.424 (3)	C18—H18A	0.93
N5—C21	1.464 (3)	C19—H19A	0.97
N5—C22	1.468 (3)	C19—H19B	0.97
C1—C2	1.528 (3)	C20—C21	1.512 (3)
C1—C6	1.537 (3)	C20—H20A	0.97
C1—H1A	0.97	C20—H20B	0.97
C1—H1B	0.97	C21—H21A	0.97
C2—C3	1.500 (4)	C21—H21B	0.97
C2—C7	1.510 (4)	C22—C23	1.510 (3)
C2—H2A	0.98	C22—H22A	0.97
C3—C4	1.522 (4)	C22—H22B	0.97
C3—H3A	0.97	C23—H23A	0.97
C3—H3B	0.97	C23—H23B	0.97
C4—C10	1.511 (4)	C24—C25	1.380 (3)
C4—C5	1.541 (4)	C24—C29	1.399 (3)
C4—H4A	0.98	C25—C26	1.388 (3)
C5—C6	1.531 (3)	C25—H25A	0.93
C5—H5A	0.97	C26—C27	1.367 (4)
C5—H5B	0.97	C26—H26A	0.93
C6—C11	1.507 (3)	C27—C28	1.385 (4)
C6—C9	1.538 (3)	C27—H27A	0.93
C7—C8	1.509 (4)	C28—C29	1.387 (3)
C7—H7A	0.97	C28—H28A	0.93
C7—H7B	0.97	C30—H30A	0.96
C8—C10	1.514 (4)	C30—H30B	0.96
C8—C9	1.536 (3)	C30—H30C	0.96
			
C29—O1—C30	118.5 (2)	N1—C11—C6	127.1 (2)
C12—N1—C11	108.77 (18)	N3—C12—N1	103.03 (19)
C12—N1—C13	119.51 (19)	N3—C12—S1	128.71 (18)
C11—N1—C13	131.62 (19)	N1—C12—S1	128.26 (18)
C12—N3—N2	113.44 (18)	C18—C13—C14	120.0 (2)
C12—N3—C19	125.4 (2)	C18—C13—N1	119.7 (2)
N2—N3—C19	121.01 (19)	C14—C13—N1	119.8 (2)
C11—N2—N3	105.01 (19)	C13—C14—C15	119.9 (3)
C19—N4—C23	114.40 (19)	C13—C14—H14A	120.1
C19—N4—C20	113.73 (19)	C15—C14—H14A	120.1
C23—N4—C20	110.29 (18)	C16—C15—C14	118.4 (3)
C24—N5—C21	116.26 (18)	C16—C15—H15A	120.8
C24—N5—C22	113.26 (18)	C14—C15—H15A	120.8
C21—N5—C22	109.82 (19)	C17—C16—C15	123.3 (3)
C2—C1—C6	109.8 (2)	C17—C16—F1	118.6 (4)
C2—C1—H1A	109.7	C15—C16—F1	118.0 (3)
C6—C1—H1A	109.7	C16—C17—C18	118.0 (3)
C2—C1—H1B	109.7	C16—C17—H17A	121
C6—C1—H1B	109.7	C18—C17—H17A	121
H1A—C1—H1B	108.2	C13—C18—C17	120.3 (3)
C3—C2—C7	109.5 (2)	C13—C18—H18A	119.8
C3—C2—C1	110.6 (2)	C17—C18—H18A	119.8
C7—C2—C1	109.6 (2)	N4—C19—N3	115.62 (19)
C3—C2—H2A	109	N4—C19—H19A	108.4
C7—C2—H2A	109	N3—C19—H19A	108.4
C1—C2—H2A	109	N4—C19—H19B	108.4
C2—C3—C4	109.2 (2)	N3—C19—H19B	108.4
C2—C3—H3A	109.8	H19A—C19—H19B	107.4
C4—C3—H3A	109.8	N4—C20—C21	111.4 (2)
C2—C3—H3B	109.8	N4—C20—H20A	109.4
C4—C3—H3B	109.8	C21—C20—H20A	109.4
H3A—C3—H3B	108.3	N4—C20—H20B	109.4
C10—C4—C3	110.0 (2)	C21—C20—H20B	109.4
C10—C4—C5	109.3 (2)	H20A—C20—H20B	108
C3—C4—C5	109.2 (3)	N5—C21—C20	110.12 (19)
C10—C4—H4A	109.4	N5—C21—H21A	109.6
C3—C4—H4A	109.4	C20—C21—H21A	109.6
C5—C4—H4A	109.4	N5—C21—H21B	109.6
C6—C5—C4	110.3 (2)	C20—C21—H21B	109.6
C6—C5—H5A	109.6	H21A—C21—H21B	108.2
C4—C5—H5A	109.6	N5—C22—C23	109.88 (18)
C6—C5—H5B	109.6	N5—C22—H22A	109.7
C4—C5—H5B	109.6	C23—C22—H22A	109.7
H5A—C5—H5B	108.1	N5—C22—H22B	109.7
C11—C6—C5	108.80 (19)	C23—C22—H22B	109.7
C11—C6—C1	111.76 (19)	H22A—C22—H22B	108.2
C5—C6—C1	108.2 (2)	N4—C23—C22	109.52 (19)
C11—C6—C9	110.99 (18)	N4—C23—H23A	109.8
C5—C6—C9	108.4 (2)	C22—C23—H23A	109.8
C1—C6—C9	108.60 (19)	N4—C23—H23B	109.8
C8—C7—C2	109.8 (2)	C22—C23—H23B	109.8
C8—C7—H7A	109.7	H23A—C23—H23B	108.2
C2—C7—H7A	109.7	C25—C24—C29	117.8 (2)
C8—C7—H7B	109.7	C25—C24—N5	123.5 (2)
C2—C7—H7B	109.7	C29—C24—N5	118.7 (2)
H7A—C7—H7B	108.2	C24—C25—C26	121.4 (3)
C7—C8—C10	110.0 (2)	C24—C25—H25A	119.3
C7—C8—C9	109.2 (2)	C26—C25—H25A	119.3
C10—C8—C9	109.4 (2)	C27—C26—C25	120.3 (3)
C7—C8—H8A	109.4	C27—C26—H26A	119.9
C10—C8—H8A	109.4	C25—C26—H26A	119.9
C9—C8—H8A	109.4	C26—C27—C28	119.5 (3)
C8—C9—C6	110.0 (2)	C26—C27—H27A	120.2
C8—C9—H9A	109.7	C28—C27—H27A	120.2
C6—C9—H9A	109.7	C27—C28—C29	120.2 (3)
C8—C9—H9B	109.7	C27—C28—H28A	119.9
C6—C9—H9B	109.7	C29—C28—H28A	119.9
H9A—C9—H9B	108.2	O1—C29—C28	123.7 (2)
C4—C10—C8	109.2 (2)	O1—C29—C24	115.7 (2)
C4—C10—H10A	109.8	C28—C29—C24	120.6 (2)
C8—C10—H10A	109.8	O1—C30—H30A	109.5
C4—C10—H10B	109.8	O1—C30—H30B	109.5
C8—C10—H10B	109.8	H30A—C30—H30B	109.5
H10A—C10—H10B	108.3	O1—C30—H30C	109.5
N2—C11—N1	109.73 (19)	H30A—C30—H30C	109.5
N2—C11—C6	123.1 (2)	H30B—C30—H30C	109.5
			
C12—N3—N2—C11	0.7 (2)	C11—N1—C12—S1	−178.65 (17)
C19—N3—N2—C11	176.39 (19)	C13—N1—C12—S1	4.6 (3)
C6—C1—C2—C3	−60.5 (3)	C12—N1—C13—C18	88.6 (3)
C6—C1—C2—C7	60.3 (3)	C11—N1—C13—C18	−87.3 (3)
C7—C2—C3—C4	−60.2 (3)	C12—N1—C13—C14	−83.7 (3)
C1—C2—C3—C4	60.6 (3)	C11—N1—C13—C14	100.5 (3)
C2—C3—C4—C10	60.1 (3)	C18—C13—C14—C15	−0.7 (4)
C2—C3—C4—C5	−59.9 (3)	N1—C13—C14—C15	171.5 (2)
C10—C4—C5—C6	−60.3 (3)	C13—C14—C15—C16	−0.2 (4)
C3—C4—C5—C6	60.2 (3)	C14—C15—C16—C17	1.5 (4)
C4—C5—C6—C11	179.3 (2)	C14—C15—C16—F1	−176.4 (2)
C4—C5—C6—C1	−59.1 (3)	C15—C16—C17—C18	−2.0 (4)
C4—C5—C6—C9	58.5 (3)	F1—C16—C17—C18	176.0 (2)
C2—C1—C6—C11	178.4 (2)	C14—C13—C18—C17	0.3 (4)
C2—C1—C6—C5	58.6 (3)	N1—C13—C18—C17	−172.0 (2)
C2—C1—C6—C9	−58.8 (3)	C16—C17—C18—C13	1.0 (4)
C3—C2—C7—C8	60.3 (3)	C23—N4—C19—N3	75.5 (3)
C1—C2—C7—C8	−61.2 (3)	C20—N4—C19—N3	−52.4 (3)
C2—C7—C8—C10	−59.4 (3)	C12—N3—C19—N4	104.2 (3)
C2—C7—C8—C9	60.7 (3)	N2—N3—C19—N4	−71.0 (3)
C7—C8—C9—C6	−59.9 (3)	C19—N4—C20—C21	−172.94 (19)
C10—C8—C9—C6	60.6 (3)	C23—N4—C20—C21	57.0 (2)
C11—C6—C9—C8	−178.1 (2)	C24—N5—C21—C20	−172.6 (2)
C5—C6—C9—C8	−58.6 (3)	C22—N5—C21—C20	57.1 (3)
C1—C6—C9—C8	58.7 (3)	N4—C20—C21—N5	−56.0 (3)
C3—C4—C10—C8	−59.0 (3)	C24—N5—C22—C23	168.36 (19)
C5—C4—C10—C8	61.0 (3)	C21—N5—C22—C23	−59.8 (2)
C7—C8—C10—C4	58.6 (3)	C19—N4—C23—C22	171.58 (19)
C9—C8—C10—C4	−61.4 (3)	C20—N4—C23—C22	−58.7 (2)
N3—N2—C11—N1	0.3 (2)	N5—C22—C23—N4	60.5 (2)
N3—N2—C11—C6	−177.88 (18)	C21—N5—C24—C25	−13.9 (3)
C12—N1—C11—N2	−1.1 (2)	C22—N5—C24—C25	114.7 (3)
C13—N1—C11—N2	175.1 (2)	C21—N5—C24—C29	165.9 (2)
C12—N1—C11—C6	177.0 (2)	C22—N5—C24—C29	−65.5 (3)
C13—N1—C11—C6	−6.8 (4)	C29—C24—C25—C26	3.0 (4)
C5—C6—C11—N2	−3.9 (3)	N5—C24—C25—C26	−177.2 (2)
C1—C6—C11—N2	−123.3 (2)	C24—C25—C26—C27	0.6 (4)
C9—C6—C11—N2	115.2 (2)	C25—C26—C27—C28	−3.1 (4)
C5—C6—C11—N1	178.3 (2)	C26—C27—C28—C29	2.0 (4)
C1—C6—C11—N1	58.8 (3)	C30—O1—C29—C28	−2.9 (3)
C9—C6—C11—N1	−62.6 (3)	C30—O1—C29—C24	176.8 (2)
N2—N3—C12—N1	−1.3 (2)	C27—C28—C29—O1	−178.5 (2)
C19—N3—C12—N1	−176.79 (19)	C27—C28—C29—C24	1.7 (4)
N2—N3—C12—S1	178.75 (16)	C25—C24—C29—O1	176.1 (2)
C19—N3—C12—S1	3.3 (3)	N5—C24—C29—O1	−3.7 (3)
C11—N1—C12—N3	1.4 (2)	C25—C24—C29—C28	−4.2 (3)
C13—N1—C12—N3	−175.31 (18)	N5—C24—C29—C28	176.1 (2)

### 3-(Adamantan-1-yl)-4-(4-fluorophenyl)-1-{[4-(2-methoxyphenyl)piperazin-1-yl]methyl}-4,5-dihydro-1H-1,2,4-triazole-5-thione (II) Hydrogen-bond geometry (Å, º)

Cg1 and Cg8 are the centroids of rings (N1–N3/C11/C12) and (C24–C29), respectively.

**Table:**

*D*—H···*A*	*D*—H	H···*A*	*D*···*A*	*D*—H···*A*
C21—H21*A*···F1^i^	0.97	2.47	3.407 (3)	162
C18—H18*A*···*Cg*1^ii^	0.93	2.81	3.661	152
C9—H9*A*···*Cg*8^iii^	0.97	2.80	3.697	155

Symmetry codes: (i) *x*, *y*−1, *z*; (ii) −*x*, −*y*, −*z*; (iii) −*x*+1, *y*−1/2, −*z*+1/2.
